# A DNA-Based Registry for All Animal Species: The Barcode Index Number (BIN) System

**DOI:** 10.1371/journal.pone.0066213

**Published:** 2013-07-08

**Authors:** Sujeevan Ratnasingham, Paul D. N. Hebert

**Affiliations:** 1 Biodiversity Institute of Ontario, University of Guelph, Guelph, Ontario, Canada; 2 Department of Integrative Biology, University of Guelph, Guelph, Ontario, Canada; Consiglio Nazionale delle Ricerche (CNR), Italy

## Abstract

Because many animal species are undescribed, and because the identification of known species is often difficult, interim taxonomic nomenclature has often been used in biodiversity analysis. By assigning individuals to presumptive species, called operational taxonomic units (OTUs), these systems speed investigations into the patterning of biodiversity and enable studies that would otherwise be impossible. Although OTUs have conventionally been separated through their morphological divergence, DNA-based delineations are not only feasible, but have important advantages. OTU designation can be automated, data can be readily archived, and results can be easily compared among investigations. This study exploits these attributes to develop a persistent, species-level taxonomic registry for the animal kingdom based on the analysis of patterns of nucleotide variation in the barcode region of the cytochrome *c* oxidase I (COI) gene. It begins by examining the correspondence between groups of specimens identified to a species through prior taxonomic work and those inferred from the analysis of COI sequence variation using one new (RESL) and four established (ABGD, CROP, GMYC, jMOTU) algorithms. It subsequently describes the implementation, and structural attributes of the Barcode Index Number (BIN) system. Aside from a pragmatic role in biodiversity assessments, BINs will aid revisionary taxonomy by flagging possible cases of synonymy, and by collating geographical information, descriptive metadata, and images for specimens that are likely to belong to the same species, even if it is undescribed. More than 274,000 BIN web pages are now available, creating a biodiversity resource that is positioned for rapid growth.

## Introduction

Most animal species await description [1] and many named taxa actually represent a species complex [2]. It has been estimated that the cost of describing all animal species will exceed US$270 billion and require centuries [3], [4]. Given this situation, it is clear that new approaches are needed to support biodiversity assessments in advance of fully developed species-level taxonomy. Biodiversity researchers have often attempted to address the taxonomic impediment in a local or regional context by assigning specimens to operational taxonomic units (OTUs) using morphological differences perceived to be indicators of species boundaries. However, it is very difficult to codify morphology-based OTUs in a format which allows their comparison among studies. The adoption of DNA sequences as a basis for OTU classification escapes this constraint; their digital nature aids the application of standardized protocols for OTU designation, the comparison of results among studies, and data preservation.

### Molecular Approaches to OTU Designation

Automated DNA-based approaches for OTU designation first saw application in ‘taxonomy-free’ groups such as bacteria [5], [6] and fungi [7], [8], but they have also proven useful for probing biodiversity patterns in animal lineages where morphology-based taxonomy is difficult [9], [10]. Although molecular analyses enable initial biodiversity evaluation in such taxa, there is no objective way to select the algorithm or input parameters that best recover actual species boundaries [11]. Instead, the microbial genomics community operates by convention; bacterial lineages with more than 3% sequence divergence at 16S rDNA are recognized as distinct OTUs [5], while the fungal community employs a 2% divergence criterion for the intergenic spacer region [8].

Because past studies of molecular biodiversity have focused on groups with incomplete taxonomy, the concordance between species diversity estimates gauged from morphology and molecules has rarely been quantitatively tested on a large scale (e.g. above the family level). Additionally, there has not been an effort to standardize protocols for the delineation of animal OTUs or to develop the registration system needed to support the comparison of results among studies. These matters are critical for any large-scale implementation of an interim taxonomic system based on DNA sequence data, but there is another requirement. For the system to support broad application, it must be based upon sequence diversity in a standard gene region(s). DNA barcoding studies on animals provide an ideal source of data because more than two million records are currently available for this 648 bp region of the cytochrome *c* oxidase I (COI) gene. Prior analysis of these data have established two important patterns: 1) More than 95% of animal species examined possess a diagnostic COI sequence array, and 2) COI divergences rarely exceed 2% within a named species, while members of different species typically show higher divergence [12], [13]. Although exceptions do occur, the presence of this ‘barcode gap’ [14] has been observed in many animal taxa [15]–[18]. Because prior studies have shown that these patterns of sequence divergence are remarkably congruent across phyla, groups with robust taxonomy can provide test sets to identify the algorithmic approach that best recognizes sequence clusters corresponding to species. The resultant algorithm can subsequently be used to analyze sequence data from groups which have seen little taxonomic investigation, illuminating species diversity in these dark taxa [19].

### Algorithms for OTU Recognition

Algorithms based on single linkage clustering [20]–[23] have been widely used to quantify microbial diversity. Blaxter et al. [24] were the first to apply this method to DNA barcode data for OTU recognition in animals, examining the impact of partitioning sequences at differing levels of sequence divergence. This approach discriminated 150 OTUs among 295 tardigrade specimens when the threshold for recognition was set at two or more nucleotide substitutions, and 121 OTUs when the criterion was raised to four or more differences. Because these tardigrades lacked species identifications, it was impossible to select the threshold that led to the strongest correspondence between OTU boundaries and actual species. Jones et al. [25] extended this approach, developing an analytical package (jMOTU) that generates OTUs using single linkage clustering with a sequence divergence threshold selected by the user. Although single linkage clustering performs well, and is computationally inexpensive, it lacks sensitivity due to chaining [26], a factor which has motivated a search for alternate approaches to OTU recognition.

Puillandre et al. [27] developed a statistical method, Automatic Barcode Gap Discovery (ABGD), to generate OTUs based on features in sequence distance distributions that indicate the presence of a ‘barcode gap’. Their method calculates distances among all pairs of sequences in a dataset and clusters them by creating a division at points where the change in slope of the distribution is highest. Partitions are recursively evaluated for division points, and splitting is sustained until all partitions possess a unimodal distribution. ABGD produces multiple possible partitioning schemes, but it is difficult to select the outcome which best recovers true species diversity without prior knowledge of the species count or without posterior examination of the alternative hypotheses with independent data. However, selection of the scheme that generated the median number of clusters has produced good correspondence in studies of real data [27].

Hao et al. [28) proposed another analytical option. Their method, Clustering 16S rRNA for OTU Prediction (CROP), employs unsupervised Bayesian clustering. Despite its name, it can be applied to sequence data from any gene through the application of a Markov Chain Monte Carlo (MCMC) search to identify partitions by optimizing a posterior probability function. Multiple parameters are available to control cluster granularity and the extent of the search for optimality. CROP uses an optimized Needleman-Wunsch [29] algorithm to perform pairwise alignments and the Quickdist [30] algorithm to generate distances. Its workflow is heavily optimized using pre-clustering and heuristics to avoid unnecessary computation.

Pons et al. [31] proposed a model-based solution, one based in phylogenetic approaches using the General Mixed Yule Coalescent (GMYC) model that represents independently evolving entities. This strategy uses a maximum likelihood approach to detect the transition of branching patterns in the gene tree from interspecific branches, following the Yule model, to intraspecific branches, following the neutral coalescent. The model optimizes the maximum likelihood value of a threshold, such that nodes in the tree above it are classified as species diversification events, following the Yule model, while those below the threshold are determined to be following the coalescent process. As such, GMYC requires prior phylogenetic reconstruction using statistically robust methods. Although GMYC has gained popularity due to its statistical robustness and accuracy [32]–[34], the high computational cost of phylogeny reconstruction and GMYC computation is a barrier to the analysis of large datasets.

### OTU Designation Through Refined Single Linkage (RESL) Analysis

The study introduces RESL, an algorithm whose design was primarily driven by the need for rapid computation to process the current 1.8 M barcode sequence records and to enable ongoing adjustments in OTU boundaries linked to the incorporation of over 10,000 new records each week. This requirement for speed and scalability set limits on the analytical options that could be considered for adoption. After reviewing prospects, RESL was developed as a staged clustering process which employs single linkage clustering as a tool for the preliminary assignment of records to an OTU and a subsequent finishing step that employs Markov Clustering (MCL), a graph analytical approach. MCL employs topological information in the similarity network along with distance values to partition a graph. It clusters records with high sequence similarity and connectivity, and separates those with lower similarity and sparse connectivity. Connectivity is explored through random walks of the network [35], a process that exposes regions of low traffic as potential cluster boundaries. True random walks are computationally expensive, but MCL [36] uses simulated walks to produce similar results at a much lower cost. The MCL method analyzes weighted graph representations of similarity networks where the graph summarizes pairwise relationships among any set of objects. Sequence data are analyzed by calculating distances between every pair of records and then constructing graphs by defining each sequence as a node and creating links between pairs of sequences whose distance is below a certain threshold. The speed of MCL and its capacity to resolve cluster boundaries beyond those achievable solely through single linkage clustering makes it a useful ‘refinement step’ in OTU designation. RESL defines the boundaries of each OTU selected for analysis by generating clusters using a range of values for the inflation parameter in MCL and then selects that which maximizes the Silhouette index [37].

### Species Recognition Through Sequence Analysis

Any algorithmic approach based on the analysis of sequence diversity in a single gene region will be an imperfect tool for the discrimination of closely related species as they will be overlooked because of their low sequence divergence. Detailed morphological, ecological, and genetic analysis can reveal such species (e.g. [38]), but these additional sources of information are not required to recognize the many species which possess deep sequence divergence from their nearest neighbour. For example, *Homo sapiens* shows 11% COI divergence from its nearest neighbour species, and most other animal species have more than 4% COI divergence from their closest relative [13], [15], [16]. Although species, such as these, with deep divergence are readily discriminated, more algorithmic finesse is required to optimize the discrimination of divergences involving young species from those resulting from intraspecific variation. Although no algorithm will be perfect, variation in performance is probable.

### Benchmarking Algorithms for the Recognition of Animal Species

**Figure 1:**
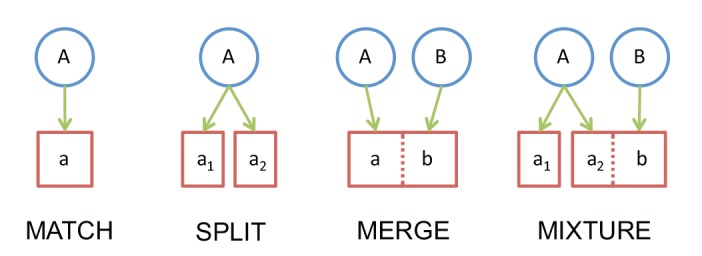
Possible patterns of association between species and BINs. Although just two species are considered, they enable illustration of the four possible patterns of association.

This study evaluates the performance of five algorithms (ABGD, CROP, GMYC, jMOTU, RESL) from two perspectives – their speed, and their effectiveness in recovering species boundaries. The speed of each algorithm was evaluated by determining the time it required to process eight trial datasets. The efficiency of each algorithm in recovering species boundaries was evaluated by examining the correspondence between the OTUs recovered by it and species memberships for each dataset. One statistical metric, F-Measure [39], was employed to quantify the ability of each algorithm to reproduce the reference groups (species in this case). Although mathematically concise, this metric has the disadvantage of being abstract and lacks a fixed scale of measurement (i.e. it can only be compared within a single dataset). As a result, performance was also evaluated by direct examination of the concordance between the OTUs established by each algorithm and recognized species boundaries. This comparison was implemented by examining the correspondence between species and OTU boundaries by placing each taxon into one of four categories: MATCH, SPLIT, MERGE, or MIXTURE. A species joined the MATCH category when all of its specimens were placed in an OTU that had no other members, while it joined the SPLIT category when it was assigned to more than one OTU that had no other members. By contrast, a species placed in a single OTU together with individuals of another species was assigned to the MERGE category. Finally, each species showing a more complex partition involving both a merge and a split was scored as a MIXTURE (Figure 1).

### Mapping Animal Diversity: the Barcode Index Number (BIN) System

Although the selection of an effective, rapid algorithm for OTU recognition is a key step in building a DNA-based registry for animal species, it needs to be coupled with a persistent informatics platform which maps each newly acquired sequence to an existing OTU or recognizes it as a founder. Ideally, each OTU should also be assigned a uniform resource identifier (URI) to enable the indexation of information on its members and integration with other data sources [40]. Finally, the system should be responsive to input from users, allowing community validation and annotation of the data associated with each OTU. The BIN system not only meets these design criteria; it also incorporates three features recognized as desirable in an interim taxonomic system – global uniqueness of names, stability of the name assigned to each specimen (or a clean audit trail), and the use of a distinctive lexicon to avoid confusion with Linnaean names [41].

This paper begins by examining the concordance between species inferred from prior morphological taxonomy and the OTUs recognized by RESL. Its speed and capacity to recover OTUs corresponding to known species are subsequently evaluated against four other algorithms. The final section of the paper describes varied aspects of the Barcode Index Number System developed within BOLD [42] to register the OTUs delineated by RESL.

## Materials and Methods

### RESL Methodology

RESL employs a staged process to assign DNA barcode sequences to OTUs. The first step involves sequence alignment; the second generates initial OTU boundaries based on single linkage clustering, and the third evaluates opportunities for refinement of OTU boundaries using Markov clustering. The final step selects the optimal partitions for OTUs based on the Silhouette index, a cluster validation method that measures how tightly clusters are integrated [37]. Uncorrected pairwise distance (p-distance) is employed for all distance calculations to avoid assumptions about the model of sequence evolution, and to maximize speed.

#### 1. Alignment

A profile Hidden Markov Model [43] of the COI protein [44] aligns the input sequences. Unlike pairwise or multiple sequence alignment methods, this approach is computationally efficient and scales linearly with the number of sequences.

#### 2. Initial Clustering

Single linkage clustering is performed on the aligned sequence data. This approach ordinarily requires the generation of a distance matrix for all pairs of sequences followed by a clustering step where sequences are grouped based on a pre-selected distance threshold [45], [46]. RESL performs distance calculations and clustering concurrently, employing the transitive property to avoid distance determinations for sequences that are certain to possess a divergence above the threshold. This strategy is implemented by flushing all clusters to disk, and retaining one or more representative sequences, depending on the diameter of the cluster, for each cluster and inter-cluster distance statistics in active memory, excepting those clusters whose members show high variability (max intra-cluster distance >2.2%, see below). The sequence divergence between each new sequence and the representative(s) of all existing clusters is then calculated. If its distance to any existing cluster is more than twice the threshold [>4.4%], it is recognized as the founder of a new cluster. If, on the other hand, it shows lower divergence, all members of the closest cluster(s) are retrieved from disk to enable more detailed analysis of sequence variation. This approach considerably reduces computational requirements without compromising accuracy, and analysis is further expedited by moving clusters to disk when they have seen no activity ( =  gained new members) for a number of cycles.

**Figure 2:**
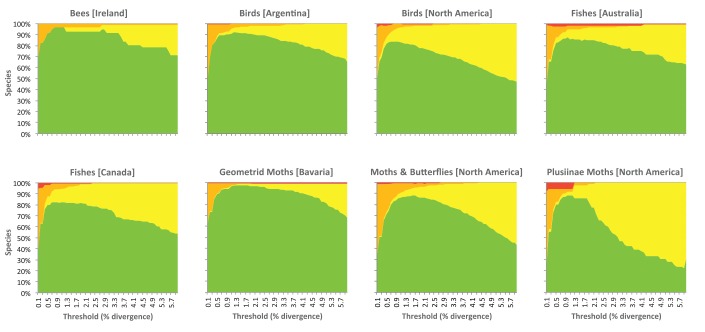
The correspondence between the species present in eight datasets and OTUs recognized through single linkage clustering with sequence divergence thresholds ranging from 0.1–6.0%. Green indicates the number of OTUs whose members perfectly match species; yellow shows those that merge members of two or more species; orange indicates cases where a species was split into two or more OTUs and red represents a mixture of splits and merges.

The implementation of single linkage clustering requires the selection of a threshold parameter, t, which represents the level of sequence divergence for the designation of OTUs. Early work [13] suggested that a threshold value of 2% was effective because most specimens showing more than this level of divergence represented different species, while those with less divergence were usually conspecific. However, this issue was examined in more detail by inspecting the patterning of OTU recovery with variance in the distance threshold for eight datasets (Table 1). Sixty single linkage cluster analyses were generated for each dataset by stepping the distance threshold parameter by an increment of 0.1% across the range from 0.1%–6.0%. The OTUs recovered at each threshold were subsequently evaluated for their concordance with recognized species boundaries (Figure 2). These analyses revealed that maximal concordance was achieved by thresholds that varied from a low of t = 0.7% (in North American birds) to a high of t = 1.8% (in Bavarian moths). It also showed that performance, as measured by the number of correctly recognized species, dropped steeply when the threshold deviated on either side of optimality. Thresholds higher than optimal inflated the number of cases where members of different species were merged in a single OTU, while thresholds lower than the optimal value increased the cases where members of what are thought by current taxonomy to be a single species were split into two or more OTUs. Based on these analyses, a threshold (t) of 2.2% was adopted as it represents the upper 99% confidence limit for the optimal thresholds in the eight test datasets 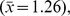 SD  = 0.40). Its adoption will lead to the merger of some distinct clusters, but such cases are addressed in the third step of the analysis.

**Table 1:** Eight datasets used to test the performance of algorithms for OTU delineation.

Datasets	GenBank Accessions	Records on BOLD	Source Publication
Birds (Argentina)	Subset of 1589 from [FJ027014: FJ028607; HM37669:HQ955631]	dx.doi.org/10.5883/DS-AVESNT1	[63]
Birds (North America)	Subset of 1936 from [DQ432694: DQ434845]	dx.doi.org/10.5883/DS-AVESNA1	[64]
Bees (Ireland)	Subset of 231 from [JQ909638: JQ909880]	dx.doi.org/10.5883/DS-BEEIRE1	[65]
Fishes (Australia)	Subset of 753 from [DQ107581:DQ108334]	dx.doi.org/10.5883/DS-FISHAUS1	[15]
Fishes (Canada)	Subset of 1359 from [EU522398: EU525162]	dx.doi.org/10.5883/DS-FISHCAN1	[66]
Geometrid Moths (Bavaria)	Subset of 649 from [GU654862:GU707400; HM37669:HQ955631]	dx.doi.org/10.5883/DS-GEOBAV1	[52]
Moths and Butterflies (North America)	Subset of 11144 from [AF549607AF549807; EF380034:EF380093; GU087155: GU439197; HQ964351: HQ964544]	dx.doi.org/10.5883/DS-LEPNA1	[67]
Plusiinae Moths (North America)	Subset of 1191 from [JN276649: JN276703; JF842288:JF860650; HQ682249:HQ971874; HM375761:HM907009; GU087601:GU803711; FJ412191:FJ412987; AF549706:AF549755; KC846141:KC846779]	dx.doi.org/10.5883/DS-PLUSNA1	N/A

#### 3. Cluster Refinement

RESL employs Markov clustering with an optimality criterion to verify and, where necessary, refine the structure of any OTU with three or more members showing some sequence variation. OTUs whose members lack sequence variation and those with just one or two members cannot be further partitioned through MCL so they are not reconsidered until their membership grows. In essence, this step examines each OTU selected for secondary analysis to determine if the MCL algorithm [36] places its members in two or more discrete sequence clusters. Under this approach, clusters whose members show high sequence variation, but lack discontinuity remain as a single OTU, while those whose sequence variation shows clear internal partitions are assigned to two or more OTUs, even if their separation is less than 2.2%. The MCL step enables the separation of sequence clusters that would be overlooked by a fixed threshold, but does not produce rampant amalgamation or fragmentation of clusters. Clusters are not at risk of merger unless they sit close to the sequence threshold. For example, if a cluster is founded by a single individual with 6.0% sequence divergence from its nearest-neighbour, there is no chance of amalgamation unless further sampling reveals an extraordinary level of variation that bridges the sequence divide. Cases do occur where specimens originally assigned to the same cluster are separated when further sampling reveals two distinct sequence clusters. In this case, the founder of the first cluster retains its membership, while an audit trail tracks information on the original cluster designation for those records that move.

The MCL algorithm delineates clusters through simulated random walks in the section of the graph surrounding each OTU selected for analysis. This walk is achieved through the repeated application of two functions, expansion and inflation, to a stochastic matrix, *M*, representing the probability of a random walker moving from one node ( =  sequence) in the graph to another. Expansion enhances traffic between nodes, while inflation raises the probability of walks within highly connected regions. The iteration of expansion and inflation ultimately results in stable segmentation of the graph. The segments present at this equilibrium point are treated as separate OTUs. Mathematical details follow:

*M* is a non-negative matrix with the property that each of its columns sums to 1. Each column *j* in the stochastic matrix corresponds with node *j* ( =  sequence *j*) of the graph. Row entry *i* in column *j* (i.e. matrix cell *M_ij_*) corresponds to the probability of walking from node *j* to node *i* (i.e. from sequence *j* to *i*). The stochastic matrix is initialized by normalizing the edge weights ( =  sequence similarity) associated with each node in the graph such that the probability of walking from node *j* to node *i* is defined by both the similarity of the two sequences and the similarity between node *j* and all other nodes.

*Expansion* involves taking the power of the stochastic matrix using the normal matrix product; in this case, squaring the matrix.

*Inflation* generates the Hadamard power of the matrix, followed by a scaling step to return matrix elements, which represent probability values, to the range of 0–1. An inflation parameter, r, is employed to tune the coarseness of the clusters. It can range from 1.0–10.0 with higher values producing finer-grained clusters. RESL optimizes the inflation parameter for each of the single linkage OTUs selected for refinement. It does this by analyzing each using MCL with r values ranging from 1.0–2.4 at 0.2 increments before selecting the value producing the highest Silhouette index.

### OTU Pipeline On BOLD

**Figure 3:**
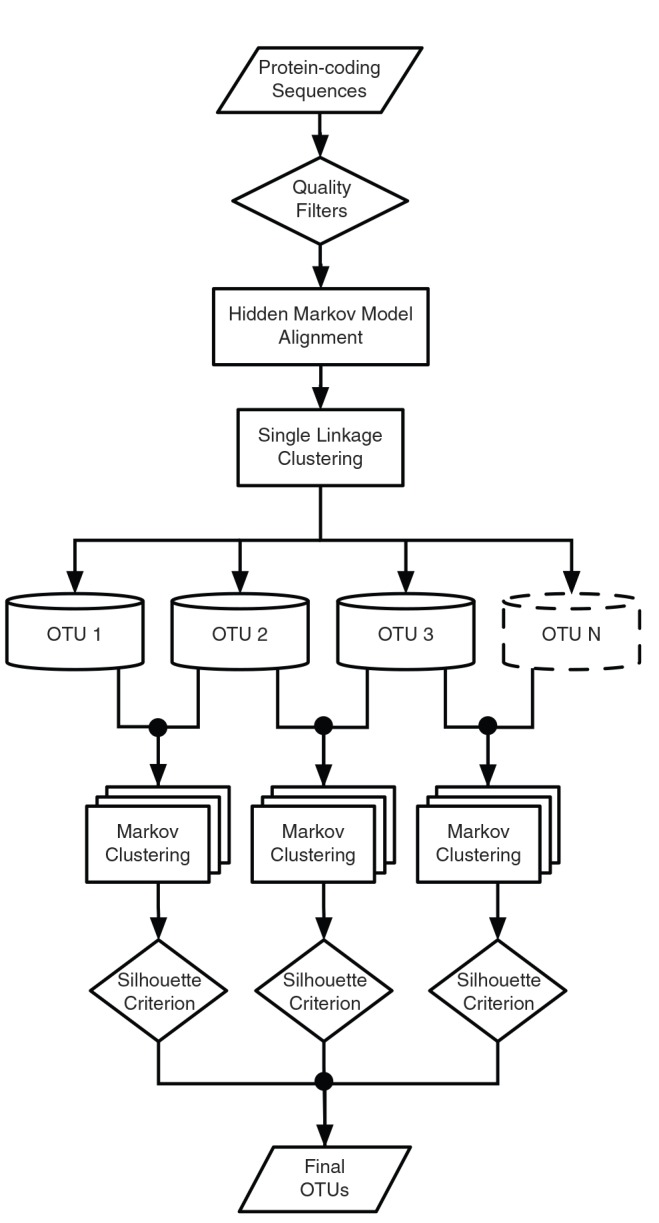
BIN pipeline for OTU generation employing RESL. OTUs initially generated through single linkage clustering are subsequently refined through Markov clustering.

A five-stage workflow on BOLD employs RESL to cluster sequences and to assign each newly collected sequence to an OTU (Figure 3).

#### 1. Quality Checks

Each new sequence is first filtered for quality, a process that excludes any record with less than 500 bp coverage for the barcode region of COI or with more than 1% ambiguous bases. If a sequence meets these quality requirements, it is then checked for reading frame shifts as indicated by stop codons or improbable peptides given the COI profile [44]. Because sequences showing these attributes are likely to derive from pseudogenes, they are excluded. Sequences are then screened to ensure that they do not derive from bacterial (e.g. *Wolbachia*) or certain external (e.g. human, mouse) contaminants by matching the sequence recovered from each specimen against a reference library of bacterial and selected vertebrate sequences. Finally, when a sequence record originates from the assembly of two or more shorter sequences, the Bellerophon package [47] is utilized to check for possible chimeras that would arise if the component sequences inadvertently (e.g. contamination, laboratory error) derived from two different taxa.

#### 2. Sequence Alignment

Each sequence that passes all quality checks is translated to amino acids and aligned to a Hidden Markov Model (HMM) of the COI protein [43]. The aligned amino acids are back translated to nucleotides to produce a multiple sequence alignment.

#### 3. Single Linkage Clustering

The next stage of analysis groups all sequences with a pairwise distance less than 2.2%, merging previously established groups when sequences are encountered that bridge a former sequence gap. The outcome is deterministic for any set of sequences, and the resulting clusters are not affected by their order of entry. Under this analytical regime, no member of any cluster is closer than the threshold (t = 2.2%) to any sequence in another cluster, but cluster diameters may be greater than the threshold.

#### 4. Markov Clustering

The cluster refinement stage takes the OTUs identified by single linkage clustering as input. When an OTU shows low distance (<4.4%) from another OTU(s), these neighbors are collapsed into a single unit before MCL clustering is used to allow more rigorous validation of their separation. MCL is run on the targeted OTUs, using inflation parameters ranging from 1.0–2.4 at intervals of 0.2, producing 8 refinement options for each OTU analyzed.

#### 5. Silhouette Criterion

The final stage takes the candidate clustering schemes generated by the 8 inflation parameter values with Markov clustering and generates a Silhouette score for each. The scheme with the maximum score is selected and reported while alternate schemes are discarded.

### Performance Comparison

Eight datasets were employed to test the performance of the five algorithms available for OTU recognition (Table 1). These datasets include four taxonomic groups (birds, fishes, moths and butterflies, bees) from two climatic regimes (temperate, tropics). Global barcode coverage is not available for any major taxonomic group, but these datasets examine taxon assemblages at both regional and continental scales. Each dataset only includes records that were associated with a valid taxonomic name; sequences associated with interim names were excluded. Seven of the datasets derive from a published study and have been placed in datasets on BOLD (Table 1), while the eighth includes new records which provide comprehensive coverage for the North American representatives of the Plusiinae, a moth subfamily (dx.doi.org/10.5883/DS-PLUSNA1 or GenBank accessions in Table 1). These eight datasets have varying sampling densities, with the average number of specimens per species ranging from 2.2 to 17.3 (Table 2). Mean intraspecific variation and nearest-neighbour distances also show substantial heterogeneity among the species in each dataset. In testing the performance of the algorithms, the taxonomic assignment for each record in these trial datasets was treated as a ‘truth’. However, it is important to recognize that these assignments may be imperfect, even for these well-studied groups.

**Table 2:** Properties of the eight datasets used in testing performance of algorithms for OTU delineation.

Datasets	Species	Sequences	Sequences per Species	Mean Max-Intraspecific Distance	Mean N-N Distance
Birds (Argentina)	497	1589	3.2	0.39	8.20
Birds (North America)	575	1936	3.4	0.43	6.70
Bees (Ireland)	56	231	4.1	0.48	8.87
Fishes (Australia)	212	753	3.6	0.50	8.73
Fishes (Canada)	190	1359	7.2	0.40	7.68
Geometrid Moths (Bavaria)	298	649	2.2	0.36	7.11
Moths and Butterflies (North America)	1327	11144	8.4	0.77	5.96
Plusiinae Moths (North America)	69	1182	17.3	0.52	3.53

The results generated by RESL were compared with those obtained through analysis of the same test datasets with the other four algorithms although GMYC was not examined for the largest dataset (because of its long run time). CROP and jMOTU incorporate sequence alignment into their clustering process, while ABGD and the phylogeny reconstruction step for GMYC require pre-aligned sequences which were generated with the MAFFT package [48]. GMYC also requires the construction of a bifurcating, ultrametric tree which was generated using BEAST [49]. Prior to phylogeny reconstruction, the most appropriate model of evolution was separately estimated for each dataset from alignments using jModelTest [50]. A GTR model with gamma-distributed substitution rates was selected for all datasets along with an estimated proportion of invariant sites. A Yule prior was used and the remaining model parameters were estimated from the data. A Bayesian search was performed on each dataset for 10 M generations logging every 1 K. The resultant logs were analyzed in TREEANNOTATER [49], selecting the tree with the maximum clade credibility while retaining node heights. The SPLITS package (http://r-forge.r-project.org/projects/splits) in R was utilized for GMYC calculations. GMYC was performed using the single-threshold strategy and default scaling parameters. Clusters were extracted from GMYC data objects using the APE package [51]. The other four algorithms require the specification of input parameter values that control the granularity of clustering. Parameters for ABGD, CROP, and jMOTU were selected to maximize the number of clusters that matched existing species (ABGD: p = 0.005, P = 0.1, n = 20, d = 1, s = 0.1; CROP: l = 0.36, u = 0.6, m = 15; jMOTU: t = 12 bp). Parameter selection for jMOTU, ABGD, and CROP was accomplished by testing a range of parameter values for each method before selecting those values that maximized the overall MATCHES across the eight test datasets. When multiple parameter values resulted in the same maximum, the values minimizing the number of MERGES, SPLITS, and MIXTURES were selected, as the performance criterion involved selection of the algorithm that best recovered the species boundaries recognized by current taxonomy, a condition that is satisfied by maximizing MATCHES and minimizing the other categories.

## Results

### RESL Performance

**Figure 4:**
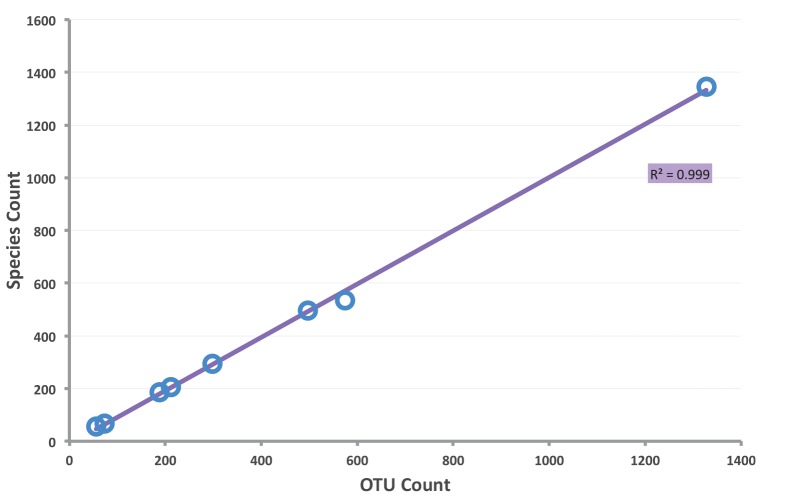
Correspondence between OTUs generated by RESL and the number of species in eight datasets.

**Figure 5:**
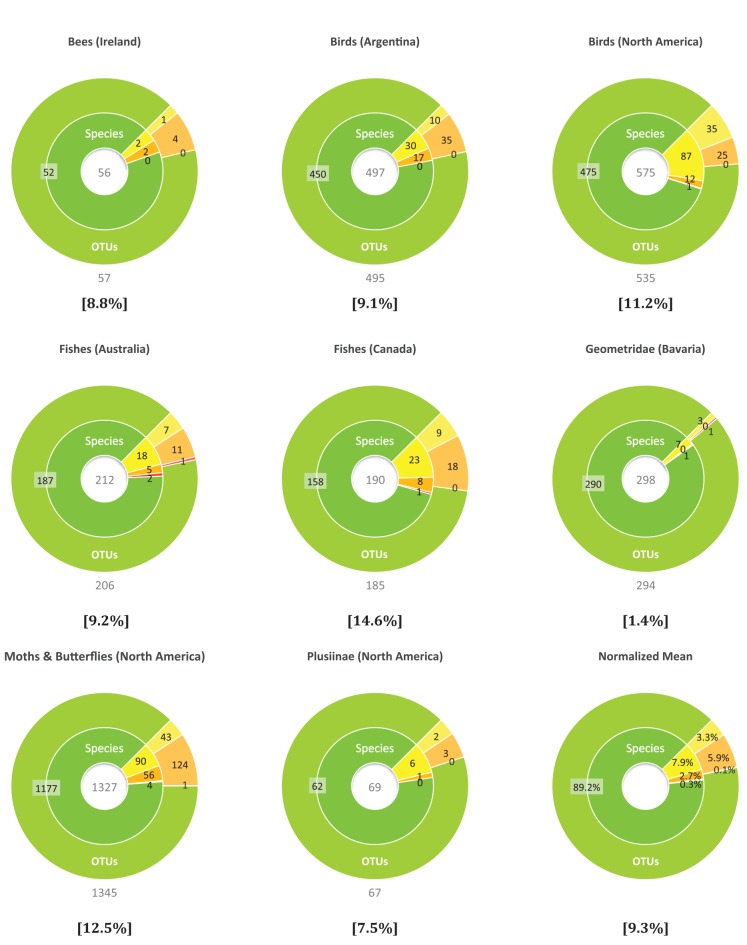
Comparison of BIN and species boundaries in eight datasets. Each inner ring partitions species, based on their assignment to BINs as MATCHES (green), MERGES (yellow), SPLITS (orange) or MIXTURES (red). Each outer ring categorizes BINs into those that MATCHED species, MERGED species, SPLIT species or MIXTURES using the same colour scheme. The number below each chart is the OTU count while the percentage indicates the incidence of OTUs that were not MATCHES.

Each of the eight test datasets was analyzed with RESL using the standard parameters (Single linkage clustering t = 2.2%; Markov clustering, r = 1.0–2.4). The OTU counts resulting from this analysis showed extremely high correlation (R^2^  = 0.999) with the number of species in the datasets (Figure 4). A more rigorous evaluation of the performance of RESL was accomplished by mapping known species onto the OTUs (Figure 5), and placing each species into one of the four categories (MATCHES, SPLITS, MERGES, MIXTURES). For example 52 of the 56 bee species from Ireland were assigned to a unique OTU (52 MATCHES), two were merged into an OTU (2 MERGES) and two other species were split into four OTUs (2 SPLITS). When viewed across the eight datasets, RESL performed well, with 89.2% of species in the MATCHES category, 2.7% in SPLITS, 7.9% in MERGES, and 0.3% in MIXTURES. The relatively high incidence of MERGES reflects the fact that RESL treats sequence divergences conservatively, pooling taxa showing low divergence rather than partitioning them. The low incidence of SPLITS in the Bavarian geometrid study reflects the fact that 14 species with the deepest intraspecific divergence were excluded from consideration in that paper to await detailed taxonomic study [52].

### Performance Comparison of Algorithms for OTU Recognition

#### Taxonomic Concordance Trials

**Figure 6:**
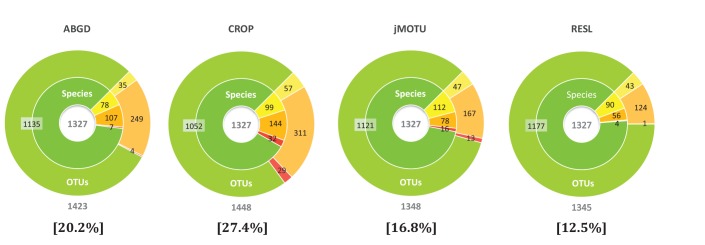
A comparison of the performance of four algorithms in OTU assignments for the Lepidoptera of eastern North America dataset. Each inner ring partitions species, based on their assignment to BINs as MATCHES (green), MERGES (yellow), SPLITS (orange) or MIXTURES (red). Each outer ring categorizes BINs into those that MATCHED species, SPLIT species, MERGED species or MIXTURES using the same colour scheme. The number below each chart is the OTU count while the percentage indicates the incidence of OTUs that were not MATCHES.

Figure 6 compares the performance of ABGD, CROP, jMOTU, and RESL in analysis of the largest dataset, the Lepidoptera of Eastern North America. Results for GMYC are unavailable because analysis was incomplete after the established time limit of two weeks. However, the performance of GMYC and RESL for other datasets is compared later. CROP, jMOTU, and RESL produced an OTU count that closely approximated the actual species number (1327), but the ABGD algorithm inflated it by about 100 species, reflecting its tendency to split sequence clusters. The tally of OTUs involved in MERGES, MIXTURES and SPLITS provides a measure of the departure of the OTUs recovered by each algorithm from recognized taxonomy. Viewed from this perspective, RESL was top performer (12.5% taxonomic discordance) for the Lepidoptera of North America dataset, while CROP was weakest (27.4%).

**Figure 7:**
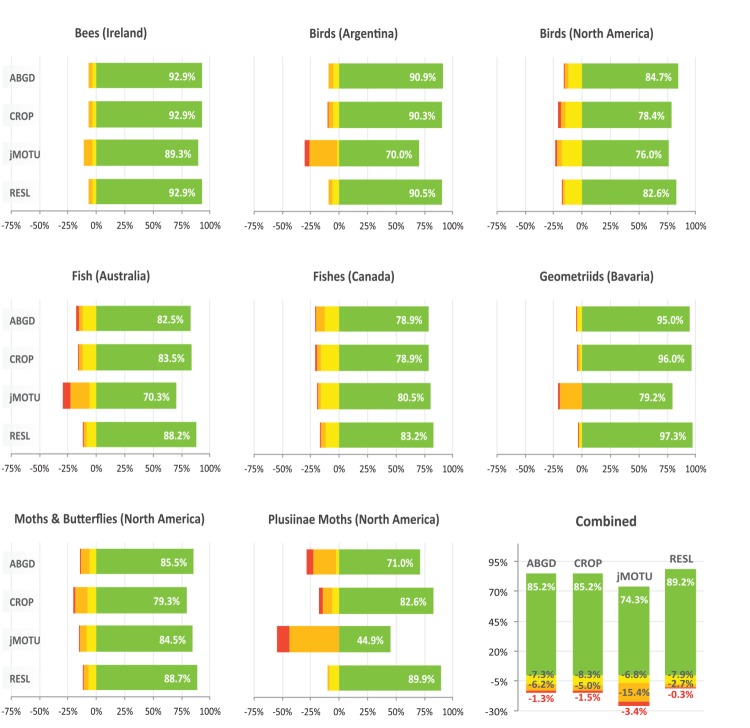
A comparison of the performance of the ABGD, CROP, jMOTU, and RESL algorithms in OTU assignments for eight datasets. Each bar consists of four categories: green – percent of MATCHES, yellow – percent of MERGES, orange – percent of SPLITS, red – percent of MIXTURES.

When this comparison was extended to all eight test datasets, RESL demonstrated the strongest performance as it either tied or achieved top ranking in MATCHES for 6 of the 8 datasets (Figure 7). On average, it scored 89.2% MATCHES versus 85.2% for ABGD, 85.2% for CROP, and 74.3% for jMOTU. In the two cases where it was not the top performer (Birds of the Argentina, Birds of North America), it was a close second. RESL performed considerably better than the other three approaches on the Plusiinae, the dataset with the largest number of specimens per species and the narrowest barcode gap (Table 2), reflecting its capacity to effectively delineate cluster boundaries in groups with low interspecific distances. RESL also showed more consistency in MATCHES (range  = 82.6–97.3%, SD  = 4.8%) than the next best result with CROP (range  = 78.4–95.6%, SD  = 6.9%). Finally, RESL showed the lowest incidence of SPLITS 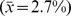 and MIXTURES 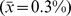 with the next best option, CROP, showing nearly twice as many SPLITS and over three times as many MIXTURES.

The performance of the four algorithms was also compared using the F-Measure [39] index which returns values from 0 to 1 with 1 indicating perfect reproduction of the ground-truth partitions (Appendix S1). RESL performed best or tied for top score in 7 of 8 datasets with this test (Table 3).

**Table 3:** A comparison of the performance of four clustering algorithms with the F-Measure.

	F-Measure			
	ABGD	CROP	jMOTU	RESL
Birds (Argentina)	0.86	0.86	0.79	0.86
Birds (North America)	0.83	0.82	0.82	0.83
Bees (Ireland)	0.92	0.92	0.90	0.92
Fishes (Australia)	0.88	0.88	0.83	0.88
Fishes (Canada)	0.94	0.96	0.97	0.95
Geometrid Moths (Bavaria)	0.71	0.71	0.62	0.71
Moths & Butterflies (North America)	0.88	0.88	0.90	0.90
Plusiinae Moths (North America)	0.87	0.90	0.85	0.93

#### Time Trials

**Figure 8:**
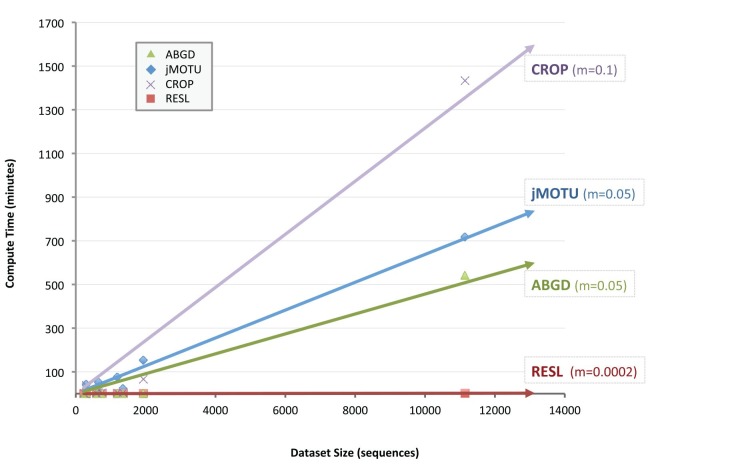
Computational time required by the ABGD, CROP, jMOTU, and RESL algorithms to generate OTUs for eight datasets.

The run-time for RESL was compared with those for the other three algorithms on a 2012 model iMac with an i7 Intel processor and 8 gigabytes of memory. CROP, jMOTU, and RESL could take advantage of the four CPU cores on this system and were allowed to do so. This analysis revealed that run times for all four algorithms rose in an almost linear fashion with increasing size of the dataset (Figure 8). However, RESL was more than 100 times faster than any of the other methods, completing the largest dataset (11.1 K sequences) in less than 2 minutes versus 541 minutes for the next fastest option (ABGD). More importantly, it showed the closest approach to linear computational complexity, a feature critical to the analyses of the barcode sequences on BOLD (1.81 M circa April 2013).

#### Performance Comparison of GMYC and RESL

GMYC differs from the other algorithms as it utilizes a model of speciation to generate OTUs and requires a phylogeny rather than sequences as input. In order to generate OTU assignments, GMYC requires time for both phylogeny reconstruction and GMYC calculation. When it was applied to the largest dataset (Lepidoptera of North America), GMYC failed to complete the phylogeny reconstruction step within the two week limit set in the study design (approximately another week would have been required to complete this step). As such, the performance of GMYC and RESL was only compared for 7 datasets.

**Figure 9:**
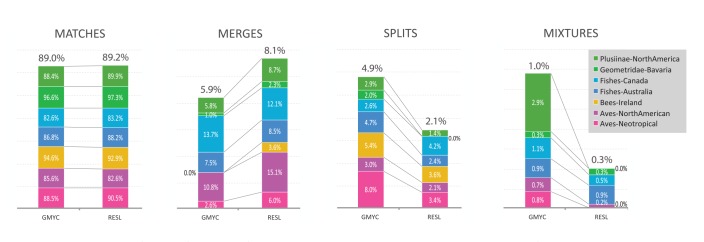
A comparison of the performance of the GMYC and RESL algorithms in OTU assignments for eight datasets. Side by side comparisons for MATCHES, LUMPS, SPLITS, and MIXTURES.

**Figure 10:**
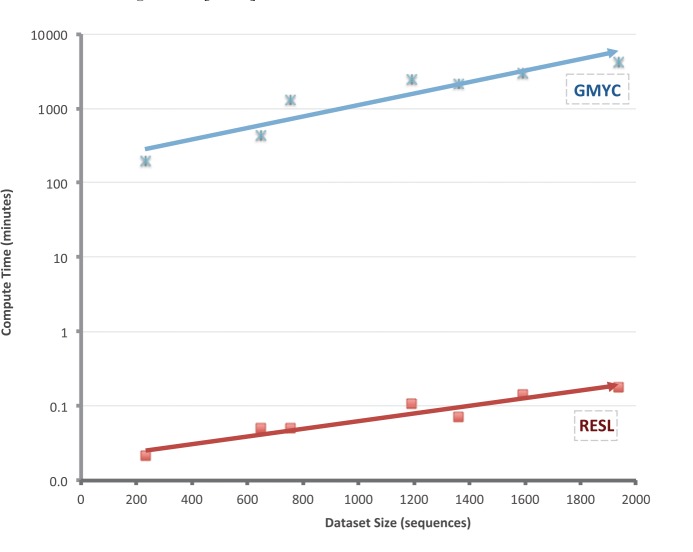
Computational time required by the GMYC and RESL algorithms to generate OTUs for eight datasets.

GMYC and RESL showed similar overall taxonomic performance (Figure 9), although RESL produced slightly more MATCHES (+0.2%), fewer SPLITS (−2.8%) and fewer MIXTURES (−0.7%), while GMYC generated fewer MERGES (−2.2%). Additionally, both algorithms showed a similar level of consistency in MATCHES (SD = 4.8%). Although their taxonomic performance was congruent, there was a dramatic difference in their run times; GMYC required 5000 times longer than RESL to complete the OTU assignments for the 7 test datasets (Figure 10).

### Implementation

**Figure 11:**
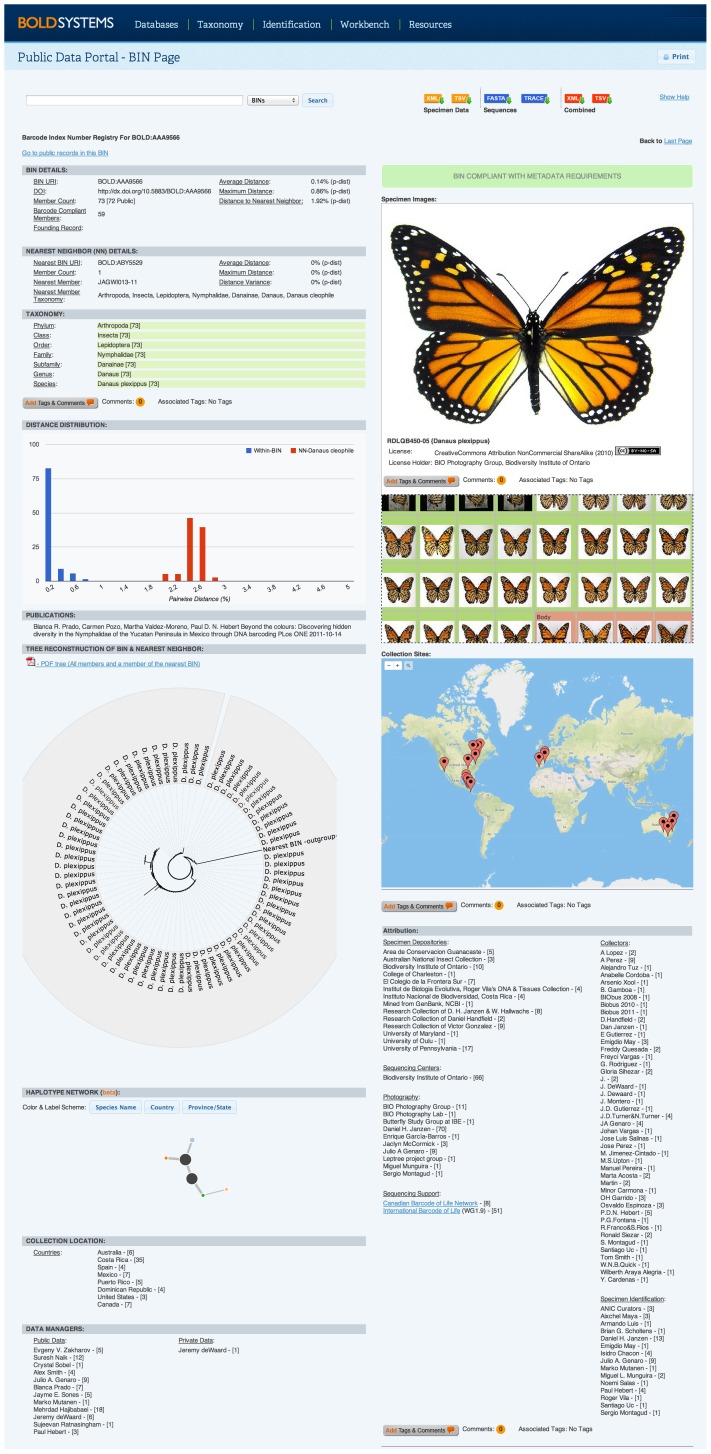
BIN page for *Danaus plexippus* (Linnaeus, 1758), the monarch butterfly.

Because of its strong taxonomic performance and speed, RESL was adopted to generate OTUs for the barcode sequences on BOLD [42]. Each of the OTUs resulting from this analysis was subsequently assigned a unique alphanumeric code with a standard structure (BOLD: 3 letters, 4 numbers). The overall informatics system supporting the indexing, storage, and retrieval of the OTUs produced through this application of RESL was termed the Barcode Index Number (BIN) System. It provides an index of unique identifiers, a database of specimens belonging to each BIN with their associated metadata, and an interface facilitating data access. The module employs Java for middleware, PHP and Javascript for the interface, and MongoDB (mongodb.org) as the database engine. The BIN pipeline analyzes new sequence data for the barcode region as they are uploaded to BOLD. Sequences that establish a new BIN add an entry to the BIN index, while sequences assigned to an existing BIN contribute their metadata to it. Each BIN is presented as a single page that exposes the aggregate data for its members. However, each BIN page holds sequence information private until data release is authorized by the submitter. Aside from revealing the gestalt of BIN pages, Figure 11 provides a sense of RESL's performance. For example, specimens of *Danaus plexippus* show just 1.88% divergence from their nearest neighbour, *D. cleophile*, but the two taxa were assigned to different BINs because of the clear break between intra- and interspecific divergence.

Because the BIN system gains power with increasing species coverage, records have been analyzed which are not fully compliant with the DNA barcode standard. Although all records in the BIN registry meet the sequence standard (>500 bp, <1% n), some lack the specimen data required to qualify for formal barcode designation. These non-compliant records are a minority because 1.68 M of the 1.81 M records derives from BOLD and nearly all (99.7%) have the required linkage to a voucher specimen and geospatial data. However, many of the 0.12 M records from GenBank lack connection to a voucher specimen and only 14.5% possess country information. BIN pages that include one or more fully compliant specimen records (sequence record >500 bp with <1% n and with trace files available, voucher specimen with at least country of origin) have been assigned a green flag, while those only based on incomplete records are marked in yellow.

The full BIN database is available at http://www.boldsystems.org/bin with an interface that supports four primary functions: search, browse, download, and annotate. Any BIN can be retrieved by its identifier or by features (geography, taxonomy, attribution metadata, literature references) associated with its members. Users can retrieve and download BIN data for any taxonomic group, or for a particular geographic region(s). When a search returns multiple BINs, users can browse the list, review summary information (taxonomy, geography, number of members) or obtain details on a particular BIN by selecting it.

#### BIN Data Model

Each BIN owes its initial establishment to a single record, but this founder is often joined through time by other sequences which match it or which show low divergence from it. The addition of each new record increases the clarity of the BIN boundaries in sequence space. BINs for common species eventually gain many records, while those for rare species may never be represented by more than one or two. As such, some BINs inevitably gain far richer metadata than others. BINs are defined by their member records and the data model reflects this by aggregating information on taxonomic assignments, points of collection, images, sequences, and attribution details for all member specimens. Key data elements include the following:

• Taxonomy: A summary of the formal and interim taxonomic assignments for all members of each BIN including higher taxonomic ranks. Each level in the taxonomic hierarchy associated with a BIN shows the number of the records attributed to a particular taxon. Cases of discordance are highlighted. Linkages between taxon names and the associated records are maintained so that taxonomic annotations made on a BIN can also be linked to individual records. It is important to emphasize that because BOLD is a workbench, the taxonomic summary includes both published and unpublished records. Users will encounter discordant taxonomic assignments, especially among unpublished records, but majority rule is a useful way to gauge the validity of a particular identification. For example, if most specimens are assigned to one species and these identifications derive from several taxonomists, this assignment is more likely to be correct than any ‘outlier’ identifications.
• Distribution: All unique sampling coordinates are gathered together with a count of the number of specimens at each site. Coordinates are linked to their original records to allow annotation.
• Images: Images for all member records are grouped by taxonomy and by orientation (e.g. ventral, dorsal).
• Sequences: Sequences are represented in three ways: 1) as a histogram of distances generated from all pairwise comparisons within the BIN together with a representative of the nearest neighbouring BIN, 2) as a neighbor-joining tree [53] and as a haplotype similarity network diagram.
• Micro-Attribution: Attribution details for each record are provided with collector, identifier, photographer, sequencing facility, and specimen depository as primary fields. Attribution is tallied and sorted based on the number of records associated with each individual or institution.

Because key elements of specimen records on BOLD, especially taxonomic assignments, are frequently revised by data providers and because of the high flow of new records, BIN metadata are dynamic. MongoDB, a document database, was adopted because of its strong ability to deal with complex, evolving data structures where updates are frequent [54].

#### BIN Identifiers

Each BIN is assigned two identifiers upon its establishment – a BOLD-generated URI and a Document Object Identifier (DOI). The URI is an internally managed alphanumeric identifier that is incremented with each new BIN. The BOLD acronym is used as a prefix for the URIs to ensure their discrimination from identifiers employed by other databases (e.g. BOLD:AAA0001 is the BIN for *Homo sapiens*). The DOI for each BIN is provided through the DataCite project (datacite.org), ensuring long-term persistence and resolution through the global registry hosted at dx.doi.org (e.g. DOI for *H. sapiens* is dx.doi.org/10.5883/BOLD:AAA0001). The assignment of a DOI also enables each BIN to gain citation through standard practices [55]. Centralized resolution of identifiers is particularly important in the case where two BINs are merged due to the submission of new sequence data which bridge a former gap or where a single BIN is split into two or more BINs when further sampling exposes a divide. In the event of a merger, following conventional taxonomic practice, the more recently registered BIN is synonymized. In the case of a SPLIT, a new BIN is established and a disambiguation option is presented. In both cases, the DOI is amended to resolve lookups to the merged BIN or to the new BIN, ensuring that the original identifiers are never lost.

#### Community Annotation

Each BIN page incorporates collateral data and metadata provided by the record providers. As well, BIN metadata can gain third-party annotation through tags which employ controlled vocabularies, and free text commentary. Annotation is dynamically linked to the primary data elements in the specimen records on BOLD, becoming a permanent part of each record. This ensures that the annotation is visible whenever the impacted data elements are displayed, maximizing the value of each annotation. These tags can be viewed as community voting tools which enable expert groups to better evaluate the accuracy of taxonomic assignments and other metadata.

#### BIN Partitions

As noted earlier, RESL merged 7.9% and split 2.7% of the species in the eight test datasets. These cases of discordance between BIN assignments and current taxonomy can have four explanations. They may reflect taxonomic error, sequence contamination, deficits in RESL, or the inability of sequence variation at COI to diagnose species because of introgression or their young age. Some discordances undoubtedly arise from taxonomic error with MERGES representing cases of overlooked synonymy, and SPLITS reflecting overlooked species. However, many MERGES have another cause; RESL fails to partition very young species because of their limited sequence divergence. Because many of these species do possess diagnostic nucleotide substitutions in the barcode region (e.g. 38), the BIN system is being extended to incorporate expert decisions in such cases. Where two or more species with diagnostic substitutions have been merged in a BIN, an expert may divide this BIN by specifying the position of the diagnostic nucleotides that allow their discrimination. These new divisions are treated as partitions of the existing BIN by extending the URI with a decimal value. For example, BOLD:AAB2314.1 and BOLD:AAB2314.2 would indicate two species of tuna, *Thunnus*, that only differ by a single nucleotide substitution in the barcode region. The use of this decimalized BIN notation has the advantage of providing a clear signal that the results of the automated BIN workflow has gained further resolution through the intervention of an expert. Moreover, each of the decimalized URIs does receive a unique DOI to allow the retrieval of information on its members.

## Discussion

This paper describes the establishment of the Barcode Index Number (BIN) system as a persistent registry for animal OTUs recognized through sequence variation in the COI DNA barcode region. Its development had two primary motivations – to enable evaluations of biodiversity patterns in advance of fully developed taxonomy and to aid taxonomic progress. It builds on prior studies which have established that most animal species show less than 2% intraspecific variation at COI, but more than 4% divergence from their nearest neighbour [13], [56]. Several earlier studies have capitalized on this pattern of sequence variation to develop algorithms for the estimation of species numbers [25], [27], [28], [31]. All do a good job, but they were designed for a different purpose – to analyze sequences resulting from a single study. As such, their scalability has not been tested, and none developed the informatics platform needed to store and compare the OTUs encountered in different studies. By contrast, this study has developed a persistent registry for OTUs, and an informatics platform enabling their storage and retrieval by expanding the capabilities of BOLD [42]. The two-algorithm process used by RESL to delineate OTUs (single linkage cluster analysis followed by Markov clustering) performed strongly, delivering OTU counts with close concordance to actual species numbers in eight datasets. However, this congruence concealed a discrepancy – just 89% showed a perfect match to a recognized species. The much closer correspondence (99%) between the species and OTU counts was a product of the merger of some species in a single OTU and the partitioning of others. These results indicate that species counts can be estimated with high accuracy through RESL, but that OTUs and species show lower overlap.

The taxonomic performance of RESL was stronger than that of ABGD, CROP, and jMOTU, but similar to that of GMYC. RESL delivered the highest incidence of MATCHES (89.2%) across the eight test datasets versus 85.2% for its closest competitor, CROP, and showed the least tendency to create SPLITS or MIXTURES. The performance of ABGD was very close to that of CROP and was slightly improved when the best partitioning scheme, of the multiple schemes output by the algorithm, was selected for each dataset, but it still delivered fewer MATCHES (86.8%) than RESL. GMYC, which could only be run on 7 of the 8 datasets, delivered a similar percentage of MATCHES as RESL, but required over 5000 times the computational effort to achieve this result. In fact, RESL generated OTU assignments 100 times more rapidly than the next fastest option. Its speed is a major advantage, enabling the 1.8 M COI sequences on BOLD to be reanalyzed every three days (on an IBM ×3650 server with 24 CPU cores and 36 gigabytes of RAM), allowing rapid adjustments in the OTU array. Based on its speed and taxonomic performance, RESL was adopted as the algorithmic approach to underpin the Barcode Index Number System, a new module on BOLD which provides a persistent registry for the OTUs which, after gaining a DOI and URI, are termed a BIN.

The eight test datasets included three that targeted a regional fauna (Bavaria, Ireland, Argentina) and five that involved continental-scale analysis. The incidence of discordances was slightly higher in the latter studies, likely reflecting the impact of regional variation in barcode sequences. Cases of discordance between BINs and accepted species boundaries merit investigation to ascertain their source. Taxonomic errors are undoubtedly responsible for some conflicts – cases of unrecognized synonymy explain some MERGES, while overlooked species create many SPLITS. Prior work has shown that the incidence of SPLITS rises with geographic scale. For example, in a study of 778 species of European geometrid moths, Hausmann [57] found that 7% of the taxa showed deep sequence divergence in local populations, but that the frequency of such cases rose to 17% when analysis spanned Europe. At least some, if not much, of this increase may involve reproductively isolated lineages that are not currently recognized as different species. Certainly, divergences at COI in excess of 2% are usually associated with reproductive incompatibility in freshwater fishes [58]. However, some SPLITS detected in our analyses likely reflect situations where intraspecific divergence is unusually high as a consequence of the inclusion of two or more phylogeographic lineages [59]. In these cases, sequence clusters assigned to different BINs actually represent a single species. Such cases create no major difficulty – the BINs can share a species name and an annotation indicating that they represent a single species. While most SPLITS likely involve overlooked species, many MERGES arise from the difficulty in diagnosing closely allied species. Cases linked to mitochondrial introgression will never be resolved through mtDNA analysis [60], but might be partitioned through the analysis of one or more nuclear genes, suggesting that one future improvement for BIN delineation in these rare cases would involve the tactical incorporation of nuclear gene information. BOLD is prepared to support an identification service based on multiple markers as the current version (v3.0) can store data on up to 150 gene regions. Although additional sequencing will be required in cases of introgression, many MERGES appear to have a simpler explanation – they involve young species that show so little sequence divergence that they cannot be separated algorithmically. However, many of these merged species do show diagnostic nucleotide substitutions in the barcode region that are correlated with the morphological or ecological traits used in species diagnosis [38]. This fact motivated the development of a decimalization option, which provides formal recognition for those BIN partitions that separate species that are too similar to gain algorithmic detection, but that possess diagnostic sequence characters in the barcode region.

The BIN system does not stand in isolation. There is an ongoing drive to improve the Linnaean taxonomic assignment for all records on BOLD that lack species-level resolution. Table 4 reveals the extent of taxonomic uncertainty surrounding current BINs; 46% lack a species designation and 8% lack a family. This taxonomic uncertainty needs to be tackled strategically with initial efforts focused on securing a family assignment for every BIN. This work can be achieved with reasonable effort and the results will be immediately useful because many biological insights accompany this increased resolution. For example, an OTU assigned to the order Diptera brings little insight beyond the fact that its members are insects with two wings. By contrast, assignment to the family Culicidae indicates an aquatic larval lifestyle, adult females which bite, and a possible role in vectoring disease. Once every BIN has been assigned to a family, efforts can be directed towards gaining generic and finally species-level resolution. This work will create a positive feedback loop because BOLD gains increasing power to place new taxa within the Linnaean hierarchy as taxonomic parameterization rises. For example, because nearly 50% of the 150 K described species of Lepidoptera now have a barcode record, BOLD is able to correctly assign most newly encountered specimens in this order to a family. Despite uncertainty in taxonomic placement, the BIN system enables examination of many issues that typically require species-level identifications. For example, it provides a powerful tool to assess local biodiversity; one recent study exploited BINs to reveal unprecedented diversity in soil mites at an arctic site [61]. Aside from enabling estimates of alpha diversity, BIN analysis permits examination of species turnover in space and time, enabling biotic change to be tracked with more precision than previously possible [62].

**Table 4:** Taxonomic information associated with specimen records and BINs on BOLD. Of the 2 M sequence records on BOLD, 1.81 M met the quality standards for a BIN assignment and they include representatives of 274 K BINs.

	BINs (274K)		SPECIMENS (1.81M)
Rank	Taxonomic Conflict	Lacking Linnaean Name	Lacking Linnaean Name
Phylum	0.0%	0.0%	0.0%
Class	0.0%	0.1%	0.3%
Order	0.1%	0.8%	1.1%
Family	1.6%	10.1%	11.7%
Genus	4.1%	23.7%	19.0%
Species	12.8%	46.0%	40.3%

Future research will undoubtedly reveal analytical approaches that are better at recognizing species boundaries from sequence information than RESL, reflecting the benefits of increasing sample sizes, and rising taxonomic and geographic coverage. Aside from general algorithmic adjustments, we anticipate that RESL will be ‘tuned’ to maximize its performance for particular taxonomic groups or environments. For example, if patterns of sequence divergence differ in systematic ways among species in different taxonomic assemblages (phylum, class), in diverse habitats (e.g. marine, freshwater, terrestrial) or among those with differing capacities for dispersal (e.g. flight, no flight), the Markov clustering step in RESL can be adjusted through modification of the inflation parameter. The prospects for future analytical improvements provide no reason to delay implementation of the BIN registry – all sequence records remain available for reanalysis. Moreover, many BIN assignments will be stable despite algorithmic adjustments because the species that they represent are deeply divergent from allied taxa. Furthermore, when adjustments are made, it will be straightforward to incorporate an audit trail tracing the past history of each BIN.

By creating a structured registry for OTUs, the BIN system provides the species-level information needed to empower biodiversity science. It delivers a much-needed identification service for the animal kingdom, breaking barriers created by the lack of specialists available to carry out routine identifications. By assigning specimens to OTUs that closely approximate species and by aggregating collateral data, the BIN system also illuminates dark taxa [19], revealing their distributions, morphologies and, as taxonomic parameterization advances, their coordinates in Linnaean space.

## Supporting Information

Appendix S1 **Cluster Accuracy Measure.** A description of the F-Measure statistic which is used to produce a single measure of concordance between the prior taxonomy and the OTUs generated by ABGD, CROP, jMOTU, and RESL.
(DOCX)
