# Supplementary material for: A DNA-Based Registry for All Animal Species: The Barcode Index Number (BIN) System
Source: PLoS One. 2013 Jul 8;8(7):e66213. doi: 10.1371/journal.pone.0066213 (PMC3704603; doi:10.1371/journal.pone.0066213)
Supplement: Appendix S1 — Cluster Accuracy Measure. A description of the F-Measure statistic which is used to produce a single measure of concordance between the prior taxonomy and the OTUs generated by ABGD, CROP, jMOTU, and RESL. (DOCX) [file pone.0066213.s001.docx]

**APPENDIX 1**: **Clustering Accuracy Measure**

In order to evaluate the quality of clustering, the result of clustering is compared against the ground truth using one of the most referenced clustering validation metrics, F-Measure (39).

The F-Measure metric combines precision and recall concepts from information retrieval.

$$Recall(i,j)=\frac{n_{ij}}{n_{i}}$$

$$Precision(i,j)=\frac{n_{ij}}{n_{j}}$$

Where n_ij_ is the number of objects of class i in cluster j, n_j_ is the number of objects in cluster j, and n_i_, is the number of objects in class i. It is defined as the combination of Recall and Precision:

$$F(i,j)=\frac{2Recall(i,j)Precision(ij)}{Precision(i,j)+Recall(i,j)}$$

Values range from 0 to 1 with a higher number indicating a closer concordance between the two clustering schemes.
